# No difference in sports participation and patient-reported functional outcomes between total knee arthroplasty and unicompartmental knee arthroplasty at minimum 2-year follow-up in a matched control study

**DOI:** 10.1007/s00167-022-07166-1

**Published:** 2022-09-26

**Authors:** Amit Meena, Elisabeth Abermann, Christian Hoser, Luca Farinelli, Caroline Hepperger, Akshya Raj, Mohit Kumar Patralekh, Christian Fink

**Affiliations:** 1 Gelenkpunkt-Sports and Joint Surgery, FIFA Medical Centre of Excellence, Olympiastraße 39, 6020 Innsbruck, Austria; 2 Research Unit for Orthopaedic Sports Medicine and Injury Prevention (OSMI), Private University for Health Sciences, Medical Informatics and Technology, Innsbruck, Austria; 3 Clinical Orthopaedics, Department of Clinical and Molecular Sciences, Università Politecnica Delle Marche, Ancona, Italy; 4 Central Institute of Orthopaedics, Vardhman Mahavir Medical College and Safdarjung Hospital, New Delhi, 110029 India

**Keywords:** TKA, UKA, Knee, Arthroplasty, Replacement, Sports, Functional outcomes, Matched control

## Abstract

**Purpose:**

The purpose of this study was to compare (1) sports participation and type of sports activity between TKA and UKA patients; (2) functional outcome and activity level between TKA and UKA; and (3) survivorship of the prosthesis in both the groups.

**Methods:**

Prospectively collected data were obtained from an arthroplasty database to identify patients who underwent primary TKA and UKA. Both the cohorts of TKA and UKA were matched, controlling for age, sex, BMI and preoperative patient-reported outcomes, which include Oxford Knee Score (OKS), Tegner activity level, and visual analog scale (VAS) for pain score. After matching the two groups, 287 TKA and 69 UKA cases were available to be included in the study. Patients were evaluated pre- and postoperatively at 2 years for sports participation and sports preference, patient-reported outcomes, activity levels, and improvement in knee pain.

**Results:**

The mean age of the TKA and UKA groups were 75.7 ± 8.1 and 74.2 ± 8.8, respectively. There was no significant difference between the two groups concerning the demographic variables. Significant improvement was noted in the weekly sports participation at the final follow-up compared to preoperative sports participation in both the TKA and UKA groups (*p* < 0.05). All patients were able to return to their desired sporting activity. No significant difference was noted between the two groups in sports participation preoperatively and postoperatively (*p* > 0.05). OKS, Tegner activity level and VAS for pain demonstrated a significant improvement from preoperative to 2 years postoperatively (*p* < 0.05). However, preoperative and postoperative patient-reported outcomes did not differ significantly between the TKA and UKA groups (*p* > 0.05). No case of revision surgery was found at a 2-year follow-up in both groups.

**Conclusion:**

Traditionally, in isolated medial compartment osteoarthritis, UKA has been considered to be the procedure with better functional outcomes, but the current study demonstrates that when confounding factors are controlled, both TKA and UKA are effective, and offer similar functional outcomes and result in similar improvement in sports participation. These findings will be helpful to counsel the patients to choose the best suitable operative procedure between UKA and TKA.

**Level of evidence:**

Level 3.

## Introduction

As many as 20% of patients who undergo total knee arthroplasty (TKA) have isolated medial compartment osteoarthritis (OA) that could be treated by either TKA or unicompartmental knee arthroplasty (UKA) procedure [1, 5]. Both TKA and UKA are effective and offer similar clinical outcomes [2]. After knee arthroplasty, patients have high expectations for the outcome and the success of the procedure is more and more evaluated by the ability to return to sports and physical activities [23]. Almost 20% of patients are not satisfied after knee arthroplasty and one of the main factors is the inability to return to the desired activity after surgery [5, 25]. Moreover, patients frequently ask the treating surgeon whether they will be able to return to sporting activity or even perform better than before surgery [15]. Therefore, patients must receive the most efficacious procedure for this condition.

Some studies [3, 6, 9, 11, 19] reported better sports participation and functional outcome in UKA compared to TKA, while other studies [8, 10] found similar results between the two groups. Although these studies [3, 6, 11, 19] were matched for demographic variables such as age, BMI, and gender, they were not matched for the preoperative patient-reported functional outcome, which may be a confounding factor for the comparison between the TKA and UKA cohorts. Therefore, it is essential to include preoperative scores along with radiographic and demographic variables for a matched analysis between the two groups.

Thus, this study aimed to analyze at 2-year after surgery: (1) weekly frequency of sports participation and preference for the type of sports activity between TKA and UKA; (2) functional outcome and activity level between the two groups; (3) survivorship of the prosthesis in both the groups. The hypothesis was that (1) both the TKA and UKA groups have a positive effect on sports participation with similar results; (2) both the groups have similar functional outcomes and activity levels; and (3) prosthesis survivorship will not be deteriorated by sports activities and survivorship will be similar in both the groups.

## Material and methods

For this retrospective study, prospectively collected data were obtained from an arthroplasty database to identify patients who underwent primary TKA and UKA. Patients were included in the study if they fulfilled the following inclusion criteria: diagnosis of primary symptomatic medial compartment knee osteoarthritis, age 50–90 years, and had a minimum of 2-year follow-up after TKA and UKA. Patients with rheumatoid arthritis, Kellgren–Lawrence grade 3–4 lateral compartment and patella–femoral OA, flexion contracture of more than 10°, varus deformity more than 10°, valgus more than 5°, preoperative flexion less than 80°, functionally deficient ACL, conditions that might interfere with the standard postoperative rehabilitation protocol and those undergoing revision knee surgery were excluded from the study.

Between January 2010 and December 2019, 385 TKA and 78 UKA were performed. Both the cohorts of TKA and UKA were matched, controlling for age, sex, BMI and preoperative patient-reported outcomes which include Oxford Knee Score (OKS), Tegner activity level, and visual analog scale (VAS) for pain score.

Two senior surgeons performed all the surgeries. TKAs were performed by medial parapatellar approach and cemented cruciate-retaining total knee prosthesis (NexGen CR, Zimmer Inc.) was used, while UKAs were performed by a limited medial parapatellar approach and Oxford mobile-bearing prosthesis (Biomet, Inc., Warsaw, IN). All patients were given a standardized postoperative rehabilitation program that consisted of a four-point gait pattern within the first 2 weeks after surgery. Crutches were used for the initial 4 weeks. For the next 8 weeks, low-impact physical activities such as walking, swimming, and static cycling were recommended. After 12 weeks, swimming, cycling, hiking, Nordic walking, and golf were allowed. According to the progress of the individual patients, further sports activities such as skiing, cross-country skiing, mountain biking, and tennis were allowed at 5–6 months.

Patients were evaluated pre-and postoperatively at 2 years for sports participation and sports preference, patient-reported outcomes, activity levels and improvement in knee pain. Preoperatively patients’ condition was evaluated 4 weeks before the surgery by questionnaire rather than immediately before surgery. Sports participation and the most frequently performed sports type were evaluated by a direct question. Patients were asked how many times in a week they participated in sports and what is the most common sports for them in the summer and winter sessions.

The study was performed at Gelenkpunkt–Sports and Joint Surgery, FIFA Medical Centre of Excellence and approved by the ethics committee of the Medical University of Innsbruck (AN2015-0050).

### Statistical analysis

Assuming the minimum clinically important difference (MCID) for OKS to be 5 points and standard deviation (SD) to be 10.24 as per the study by Beard et al. [2], the sample size necessary was 56 in UKA and 280 in TKA groups, as calculated by G Power software for Mann–Whitney *U* test, with 90% power and significance level of 0.05 and an allocation ratio of 5. The current study had a sample of 69 patients in the UKA and 287 in the TKA group.

Categorical data were summarized by number (%) and continuous data by median (range, IQR) or mean (SD) for non-normal and normally distributed data, respectively. Shapiro–Wilk test was used to access the normality of continuous data. All continuous variables were found to be non-normal, indicating an appropriate nonparametric approach. The Chi-square test was used for categorical data (gender and frequency of weekly sports) as appropriate. Wilcoxon sign rank tests were used for comparing pre- and postoperative data and the Mann–Whitney *U* test for numerical data between TKA and UKA groups. All statistical analyses were performed using SPSS (Version 28.0.1, IBM Corp.) and a *p* value of < 0.05 was considered significant.

## Results

After matching the two groups, 287 TKA and 69 UKA cases were available to include in the study. The mean age of the TKA and UKA groups was 75.7 ± 8.1 and 74.2 ± 8.8, respectively. Demographic details of both groups are given in Table 1. There was no significant difference between the two groups concerning the demographic variables (Table 1).

**Table 1:** Comparison of the demographic characteristics between TKA and UKA

Demographic characteristics	TKA (*n* = 287)	UKA (*n* = 69)	Total (*n* = 356)	*p* value
Gender				
Female	141 (49.1%)	27(39.1%)	168	0.135 ^b^
Male	146 (50.9%)	42(60.9%)	188	
Age(years)				
Mean ± SD	75.7 ± 8.1	74.2 ± 8.8	75.4 ± 8.2	0.208^a^
Median (IQR)	76 (10, 71–81)	74 (11, 69–80)	76 (10, 71–81)	
Range	49–90	48–90	48–90	
Body-mass index (BMI)				
Mean ± SD	26.5 ± 3.8	26.8 ± 3.2	26.6 ± 37	0.399^a^
Median (IQR)	26.0 (4.2, 24.2–28.4)	26.4 (4.1, 24.6–28.7)	26.0 (4.3, 24.2–28.5)	
Range	18.5–38.9	21.5–35.7	18.5–38.9	

^a^Mann–Whitney *U* test

^b^Chi square test

**Fig. 1:**
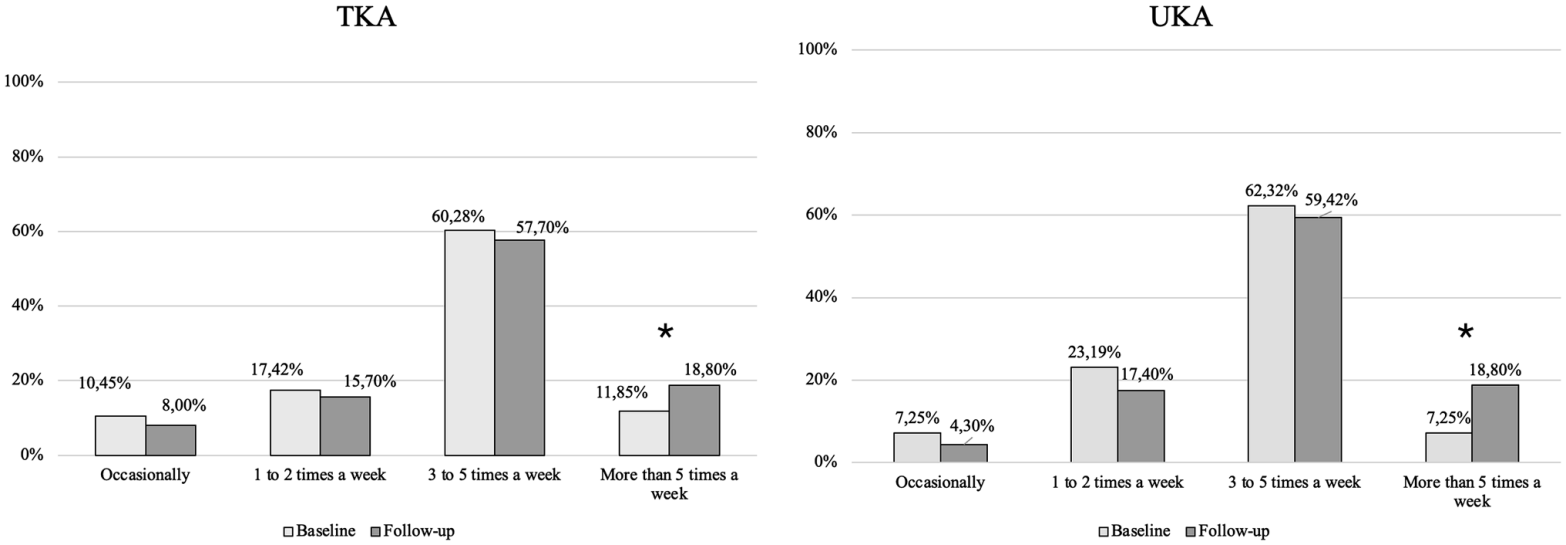
Comparison of sports participation between TKA and UKA preoperatively and at the 2-year follow-up. *Significant difference (*p* < 0.05)

Significant improvement was noted in the weekly sports participation at the final follow-up compared to preoperative sports participation in both the TKA and UKA groups (*p* < 0.05). All patients were able to return to their desired sporting activity. No significant difference was noted between the two groups in sports participation preoperatively and postoperatively (*p* > 0.05) (Table 2 and Fig. 1). Following TKA, the most common sports practiced were hiking, cycling, and swimming in order of decreasing frequency in summer; while in winter the most commonly practiced sports were skiing, followed by cross-country skiing and hiking. A similar order of sports activities was noted in the UKA group during the summer and winter sessions.

**Table 2:** Comparison of sports participation of study subjects preoperatively and at the 2-year follow-up

Sports participation	Preoperative		24 months	
	TKA (*n* = 287)	UKA (*n* = 69)	TKA (*n* = 287)	UKA (*n* = 69)
Occasionally	30 (10.5%)	5 (7.3%)	23 (8.0%)	3 (4.3%)
1–2 times a week	50 (17.4%)	16 (23.2%)	45 (15.7%)	12 (17.4%)
3–5 times a week	173 (60.3%)	43 (62.3%)	165 (57.7%)	41 (59.4%)
More than 5 times a week	34 (11.9%)	5 (7.3%)	54 (18.8%)	13 (18.8%)
*p* value	0.442^a^		0.762^a^	

(*n*) = number of patients

^§^Chi square test

**Fig. 2:**
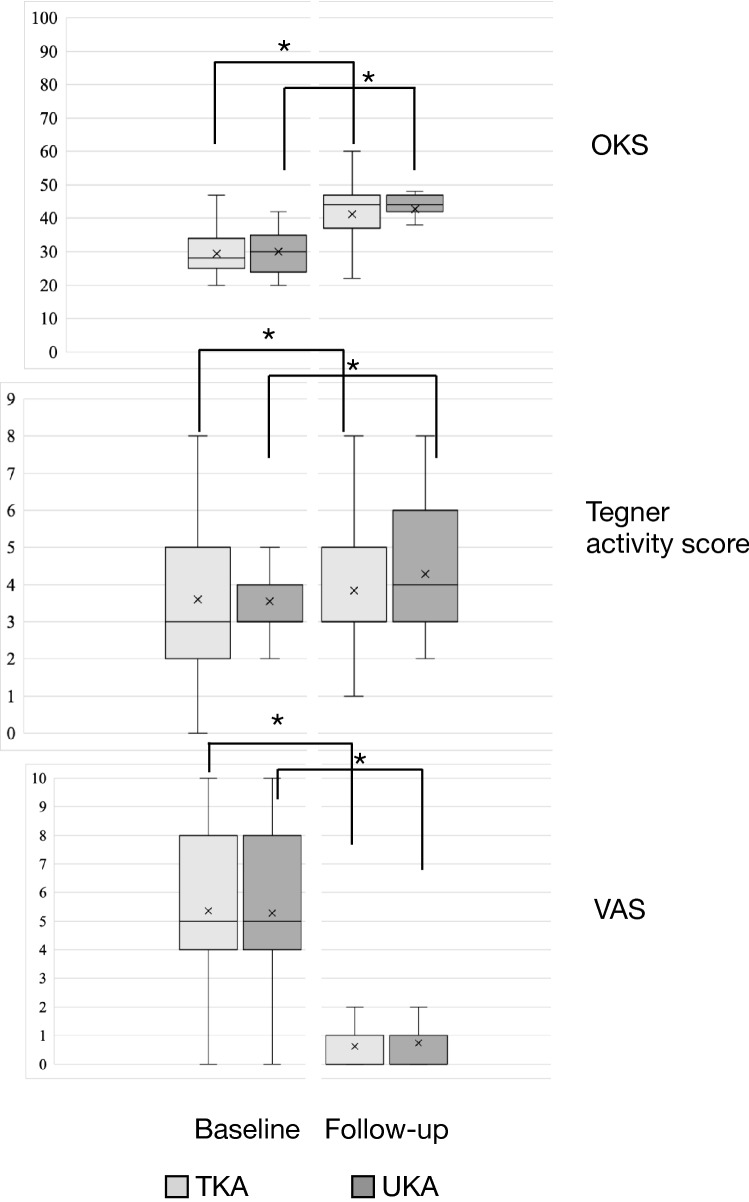
Comparison of OKS, Tegner activity score, and VAS between TKA and UKA preoperatively and at the 2-year follow-up. *Ssignificant difference (*p* < 0.05)

Preoperative and postoperative OKS did not differ significantly between the two study groups (*p* > 0.05). However, both the groups demonstrated a significant improvement in OKS from preoperative to 2 years postoperatively (*p* < 0.05). Similarly, Tegner activity level significantly improved from preoperative to postoperative in both the groups (*p* < 0.05), but no significant difference was noted in both the groups at the final follow-up. Significant improvement in knee pain after surgery was reported in both groups (*p* < 0.05). However, the difference between the TKA and UKA groups with respect to VAS for pain at 2-year follow-up was not significant (*p* > 0.05) (Table 3 and Fig. 2). No case of revision surgery for any reason was noted in either group at the 2-year follow-up.

**Table 3:** Comparison of Oxford Knee Score, Tegner activity score, and VAS for pain score between TKA and UKA

Variables	TKA (*n* = 287)	UKA (*n* = 69)	Total (*n* = 356)	*p* value
Oxford Knee Score				
Preoperative				
Median (IQR, 25th–75th percentile)	28 (9, 25–34)	30 (11, 24–35)	28 (9.5, 24.8–34)	0.376^a^
Range	27 (20–47)	22 (20–42)	27 (20–47)	
At 24 months				
Median (IQR, 25th–75th percentile)	44 (9.5, 37.5–47)	44 (5, 42–47)	44 (8.3, 38.8–47)	0.472^a^
Range	48 (12–60)	31 (17–48)	48 (12–60)	
Intragroup *p* value	< 0.001^b^	< 0.001^b^	< 0.001^b^	Improvement in OKS 0.742^a^
Tegner activity score				
Preoperative				
Median (IQR, 25th–75th percentile)	3 (3, 2–5)	3 (1, 3–4)	3 (3, 2–5)	0.928^a^
Range	8 (0–8)	8 (0–8)	8 (0–8)	
At 24 months				
Median (IQR, 25th–75th percentile)	3 (2, 3–5)	4 (3, 3–6)	3 (2, 3–5)	0.622^a^
Range	7 (1–8)	7 (1–8)	7 (1–8)	
Intragroup *p* value	0.008^b^	0.005^b^	0.002^b^	Improvement in TAS 0.655^a^
VAS for pain score				
Preoperative				
Median (IQR, 25th–75th percentile)	5 (4, 4–8)	5 (4, 4–8)	5 (4, 4–8)	0.826^a^
Range	10 (0–10)	10 (0–10)	10 (0–10)	
At 24 months				
Median (IQR, 25th-75th percentile)	0 (1, 0–1)	0 (1, 0–1)	0 (1, 0–1)	0.063^a^
Range	9 (0–9)	6 (0–6)	9 (0–9)	
Intragroup *p* value	< 0.001^b^	< 0.001^b^	< 0.001^b^	Reduction in VAS 0.524^a^

^a^Mann–Whitney *U* test

^b^Wilcoxon signed rank test

## Discussion

The most important finding of this study was that following TKA and UKA, sports participation significantly improved in both groups. The frequency of sports participation was similar between the two groups. All the patients continue to practice their desired sports activities. Patient-reported functional outcomes: OKS, Tegner activity level and VAS for pain, significantly improved from the preoperative level; however, no significant difference was noted at the 2-year follow-up between the TKA and UKA groups. No case of revision surgery for any reason was noted in either group at the 2-year follow-up.

After matching for demographic factors and preoperative function, the present study found that sports participation significantly improved and both UKA and TKA patients continued their sports participation successfully. Both groups did not differ in postoperative sports participation. This finding is contrary to the result of previous studies [3, 5, 6, 24], where the authors found better postoperative sports participation in the UKA group than in TKA. In their study, Harbourne et al. [5] noted that the UKA group was more likely to return to desired sports activities than the TKA group. However, this study was not matched control, and preoperative functional outcomes were worse in TKA than in UKA. Moreover, the TKA group was older and had a higher BMI than the UKA group. Age is a negative predictor of postoperative activity level [4, 14] and it is worth noting at this juncture that patients undergoing UKA tend to be younger than those undergoing a TKA [13]. Hence, matching for age becomes important for unbiased comparison between UKA and TKA. Similarly, in other studies [3, 6], a higher activity level was reported in UKA compared with TKA in comparable patients. Although in these studies both the groups were matched for age, sex and BMI, they did not match the preoperative functional outcomes. Witjes et al. [24] found in their systematic review that patients were able to return to both low- and higher-impact sports in both TKA and UKA, but sports participation was more after UKA than TKA. Again, included studies in this systematic review were not matched controlled. Therefore, lower sports participation in TKA than in UKA was not a true picture of outcomes in the previous studies.

Waldstein et al. [21] in their systematic review on sport and physical activity after UKA showed that the frequency of sports per week either increased or remained unchanged and the most popular activities after surgery were hiking, cycling, and swimming. In their recent study, Meena et al. [14] reported increased sports activity after TKA at mid-term follow-up. They found that the frequency of weekly sports activities significantly increased after surgery and the most common sports in summer were hiking, cycling, and swimming, and in winter skiing, followed by cross-country skiing and hiking. Hepperger et al. [7] found that sports activities were increased or maintained after TKA and the common sports in summer were hiking, cycling, and swimming, and in winter skiing, hiking and cross-country skiing. Similarly, in their online survey of 120 European Knee Association (EKA) members, Thaler et al. [18] recommended aerobics, hiking, and Nordic walking after 12 weeks of TKA. At 6 months, they additionally recommended mountain biking/incline cycling and skiing sports. In the current study, a greater proportion of patients practice sports more than five times a week postoperatively and the most common summer and winter sports align with previous literature.

In the current study, the OKS, Tegner activity level, and VAS for pain score significantly improved in both the UKA and TKA groups and there was no difference between the groups. This is in agreement with previous literature. In their large cohort study, Lyons et al. [13] found that the improvement in functional scores was similar between the TKA and UKA groups. A recent randomized controlled trial [2] also found no difference in the OKS between TKA and UKA groups at 5 years of follow-up. Similarly, in another study [17], the authors found comparable improvement in functional outcomes in both the groups after arthroplasty. In their systematic review, Kleebad et al. [12] found higher OKS, Hospital for Special Surgery (HSS) score and Western Ontario and McMaster Universities Osteoarthritis Index (WOMAC) scores for UKA, but Knee Society Score (KSS) was equivalent between UKA and TKA. Improvement in VAS for pain was also in agreement with previous literature. In their meta-analysis, Wilson et al. [22] found similar postoperative pain scores for both the TKA and UKA groups.

On the contrary, some previous demographically (age, sex, BMI) matched control studies [3, 6, 19, 20] showed better functional outcomes in UKA compared to TKA. This does not paint the full picture, as these studies were not matched for preoperative functional scores and preoperative scores were found to be higher in the UKA group, which resulted in more improvement in UKA than in the TKA group. A higher preoperative functional score was found to be predictive of better postoperative scores [5, 16]. Similarly, a skew of postoperative patient-reported outcomes in favor of UKA in the previous systematic reviews and meta-analysis was due to the fact that the included studies were not matched for demographic factors and/or preoperative functional scores with TKA cohorts. None of the patients in either group required revision in the current study; however, owing to the short-term follow-up, long-term survivorship was out of the scope of the study.

There are a few limitations of the study. First, this was a retrospective analysis of patient-reported functional outcomes; however, all data were collected prospectively. A large cohort randomized control trial should be conducted, which will be of higher evidentiary value. Second, both the groups were matched control; therefore, the overall sample size was reduced and matching might result in selection bias. Third, with short-term follow-up, it was not possible to analyze the influence of the sports activity on the longevity of the implant; therefore, a long-term follow-up study comparing both the groups for revision and the effect of sports activity on prosthesis was required. The fourth limitation is the smaller sample size in the UKA group; however, the indications for a UKA are limited compared to TKA and hence one would expect the UKA group to be smaller in number.

Despite the limitations, to the best of the authors’ knowledge, this is the first study where matching of both UKA and TKA groups was done for preoperative functional scores in addition to demographic parameters, which offers a proper control group for comparison and eliminates the confounding factors.

The current study has clinical relevance to managing the expectations of patients with medial unicompartmental OA regarding the level of sports activity. With these findings, surgeons will be able to counsel their patients to choose the best suitable operative procedure between UKA and TKA regarding postoperative activity level. A case-specific approach would be prudent when considering the surgical management of medial OA of the knee in active patients.

## Conclusion

Traditionally, in isolated medial compartment osteoarthritis, UKA has been considered to be the procedure with better functional outcomes, but the current study demonstrates that when confounding factors are controlled, both TKA and UKA are effective, and offer similar functional outcomes and result in similar improvement in sports participation. These findings will be helpful to counsel the patients to choose the best suitable operative procedure between UKA and TKA.
